# Psychotic due to bath salts and methamphetamines: emergency cesarean section under general anesthesia

**DOI:** 10.7555/JBR.32.20180023

**Published:** 2018-05-15

**Authors:** Nina Schloemerkemper

**Affiliations:** Department of Anesthesiology and Pain Medicine, University of California Davis, Sacramento, CA 95817, USA.

**Keywords:** bath salts, psychoactive substance, legal high, designer drug, methamphetamine, general anesthesia, cesarean section

## Abstract

The use of “bath salts” or other new psychoactive substances, otherwise known as “legal highs”, is increasing. Illicit drug use during pregnancy is not uncommon. Nevertheless, literature reporting bath salts and their effect on pregnancy is scant. Besides, there seems to be no literature about bath salts and conduct of general anesthesia. This case report describes a general anesthetic for the surgical delivery of an infant to a woman under the acute influence of bath salts and methamphetamines.

## Introduction

In pregnancy, the prevalence of illicit drug abuse is estimated to be around 11% with marijuana being the most frequent used^[1]^. There is no data about the prevalence of bath salt use during pregnancy. Literature about the effects of psychoactive drugs during pregnancy is limited^[2]^. In addition, there is no published information to date about general anesthesia in patients under the influence of bath salts. The abuse of bath salts is increasing^[2^‒^5]^. In the United Kingdom, a prevalence of at least 3% was reported in the 16‒24 year age group^[4]^. The US National Institute on Drug Abuse published data indicating that approximately 0.8% of 12^th^ graders abuse bath salts (https://www.drugabuse.gov/drugs-abuse/synthetic-cathinones-bath-salts). New psychoactive substances seem to be most commonly used by individuals also involved with other substance abuse^[4]^. In addition, bath salts are frequently mixed in with other drugs like 3,4-methylenedioxymethamphetamine (MDMA, commonly known as ecstasy) (https://www.drugabuse.gov/national-survey-drug-use-health). Gaps in knowledge about new psychoactive substances and the need for research have become apparent^[4^,^6]^.

This report describes a successful general anesthetic for an uncooperative and violent patient under the influence of methamphetamines and bath salts presenting for cesarean section.

## Case report

Our patient in her early 20’s had a history of substance-induced psychosis. She had used methamphetamines for several years and admitted continued use throughout pregnancy. She presented near term with premature rupture of membranes, and was clearly psychotic as well as verbally and physically abusive. The patient admitted recent use of methamphetamines which was confirmed in a urine toxicology screen. Because of concern for self-harm, she was placed on involuntary psychiatric hold. On exam, the fetus was found to be in breech presentation. Considering the acute intoxication (at that time thought to be solely due to methamphetamines), aggressive behavior and paranoid delusions, the decision was made to perform a cesarean section rather than attempting external cephalic version followed by induction of labor. A relative was identified as surrogate decision maker, who agreed with the plan for a cesarean section under either general or regional anesthesia. During reassessment, the patient was increasingly agitated and threatening. In order to protect her own safety as well as that of the fetus, the decision was made to perform a cesarean section immediately.

The patient’s aggression made restraint by security personnel necessary for placement of an intravenous cannula. Immediately, 2 mg of midazolam were given, followed by incremental injection of a total of 120 mcg of dexmedetomidine over approximately 8 minutes. The patient became less agitated and appeared intermittently sleepy. Nevertheless, she still displayed bursts of aggression and required continued restraints on her way to the operating room and prior to induction.

Because of the patient’s physical outbursts, it became clear that regional anesthesia was not an option and general anesthesia was chosen. Routine anesthesia monitors were used. The baby’s status was unknown because the aggression and then time constraints made it impossible to apply fetal monitors. The first recorded vital signs of the mother in the operating room were a blood pressure of 124/88 mmHg and a pulse of 86. After skin preparation, draping and pre-oxygenation, general anesthesia was induced with 400 mg (5.5 mg/kg) propofol and succinylcholine. A higher than usual induction dose of propofol was chosen as the patient was still hypervigilant during pre-oxygenation. Tracheal intubation was uneventful. The patient was hemodynamically stable and anesthesia was maintained with inhalational sevoflurane in O_2_/N_2_O at standard end-tidal levels.

A low-transverse cesarean section was performed. The amniotic fluid was clear and a vigorous baby was delivered one minute after incision. Apgar scores were 6 and 8 at 1 and 5 minutes, respectively. A cord blood gas unfortunately clotted.

After administration of pitocin (rate: 30 IU over 1 hour), methergine 0.2 mg intramuscular (IM) and hemabate 250 mcg IM, and the placement of a B-Lynch suture the uterine tone was adequate. The overall estimated blood loss was 800 mL.

In addition to the medication mentioned above, the patient received midazolam 2 mg, fentanyl 150 mcg, ketorolac 30 mg, hydromorphone 2 mg and lactated Ringer’s 1,500 mL solution after delivery of the baby. The patient was slow to wake up from anesthesia but was successfully and uneventfully extubated approximately 70 minutes after surgery end with additional personnel in attendance due to concern for repeated aggression. Fortunately, this was not the case and the post-partum course was uneventful as her psychosis resolved. On further questioning, the patient admitted to having been high on bath salts in addition to methamphetamines.

## Discussion

Bath salts, or “synthetic cathinones”, are a subgroup of “new psychoactive substances” that are also called “designer drugs”. Originally derived from the *Catha edulis* (Khat) plant, they are β-keto analogues of amphetamines^[5^,^7]^. Bath salts act by inhibiting re-uptake or increasing release of noradrenaline, dopamine, and serotonin to various degrees^[7]^. The signs and symptoms of bath salt intoxication are nonspecific, although agitation, psychosis and violent behavior are common^[5^,^7^‒^8]^. Symptoms can be mild, but serious clinical courses have been described including seizures, respiratory failure, hyperpyrexia, compartment syndrome, rhabdomyolysis, renal failure, disseminated intravascular coagulation, and even death^[4^‒^5^,^7]^. Urine toxicology screening as well as gas chromatography and immunoassays are possible diagnostic tools although impractical: the results take several days to come back^[6^‒^7^,^9]^. Clinicians therefore rely on the patient’s history. Unfortunately, most often patients are not asked about the use of bath salts^[10]^. Our patient was not specifically asked either and only revealed the information once the acute phase of her intoxication had passed. At the time of her emergency surgery, we were therefore unaware of the same day use of bath salts.

Sedation of a combative patient may be necessary. However, drugs commonly used can cause hemodynamic instability and hypoventilation, both of which can impair placental blood flow and oxygen transfer to the fetus. Although the use of benzodiazepines has been reported in acute intoxications^[7]^, we were hesitant to give more benzodiazepines due to concern for respiratory depression during transfer to the operating room. One concern was also the respiratory effort of the newborn. In a prior case report, 0.025 mg/kg of midazolam given intravenously before a cesarean section did not have any adverse effects on the newborn^[11]^. Our patient received 2 mg of midazolam prior to the cord being clamped. This dose was slightly higher (0.028 mg/kg) than in the study mentioned above but did not seem to have an obvious negative impact on the newborn.

In order to minimize midazolam administration, we used dexmedetomidine as an adjunct. Considering that acute consumption of methamphetamine and bath salts causes an increase in sympathetic activity, an alpha2 receptor agonist may be a logical choice. A placental transfer rate of 0.76 shows that dexmedetomidine does cross the placenta but had no negative impact on APGAR scores^[12^‒^13]^.

This patient received an unusually large dose of propofol for induction. This was an *ad-hoc *decision by the anesthesia provider due to the apparent resistance of the patient to the sedative medication that had already been administered.

In patients known to have ingested bath salts, it has been recommended to avoid the administration of fentanyl due to possible serotonergic effects^[7]^. This theoretical interaction was not supported by our clinical experience.

Special attention should be placed on the potential for rhabdomyolysis, hyperthermia and hyponatremia that can occur during intoxication^[7]^. It would have been clinically informative to obtain a maternal arterial blood gas as the ingestion of synthetic cathinones has been associated with acidosis in 43% of patients^[14]^.

## Conclusion

Drug abuse during pregnancy is not uncommon. Health care providers are commonly not aware which substances their patients are using. Unfortunately, timely screening tests for bath salts are not available^[9]^, making diagnosis difficult. Due to their increased use, bath salts should be considered in the differential, particularly if a patient presents in a psychotic and aggressive state^[2]^.

While literature is lacking regarding the newer recreational drugs and their interaction with anesthetic drugs, we present a successful general anesthetic for an uncooperative and violent patient under the influence of methamphetamines and baths salts presenting for cesarean section. The patient was hemodynamically stable and the baby was born with APGAR scores of 6 and 8 suggesting successful management of mother and fetus in the difficult setting of unknown substance abuse.
